# Splenic and Portal Vein Thrombosis With Concurrent Splenic Infarction: A Rare Manifestation of Essential Thrombocythemia

**DOI:** 10.7759/cureus.15768

**Published:** 2021-06-20

**Authors:** Manisha Gulia, Neha Sharma, Yajur Arya, Arshi Syal, Monica Gupta

**Affiliations:** 1 Internal Medicine, Government Medical College and Hospital, Chandigarh, IND

**Keywords:** essential thrombocythemia, mesenteric venous thrombosis, splenic infarction, portal vein thrombosis, myeloproliferative disorder

## Abstract

Mesenteric venous thrombosis (MVT) presents with a wide range of clinical presentations depending on the vessel involved, degree of thrombosis, and the extent of bowel wall ischemia. MVT usually has an insidious presentation and is often a forerunner of an underlying disorder. Essential thrombocythemia (ET) presenting itself as MVT along with splenic infarction is a rare presentation. Here, we report the case of a 54-year-old female with massive splenomegaly, thrombocytosis, and acute splenic and portal venous thrombosis along with multiple splenic infarcts. Bone marrow suggested ET with JAK2V617F mutation positivity. She was managed conservatively and made an uneventful recovery.

## Introduction

Essential thrombocythemia (ET) is an acquired myeloproliferative disorder (MPD) characterized by a persistent elevation of platelet count. The annual incidence has been estimated between 0.77 and 2.53 per 100,000 people and is comparably more common in young females [1]. The clinical manifestations range from vaso-occlusive events to bleeding manifestations and transformation into leukemia or other myeloid neoplasms at later stages. Thrombosis at unusual sites is rare and happens to be a severe complication of ET [1]. Around 8-15% of the cases are idiopathic and the remaining have a direct relation with various conditions, particularly MPD [2]. Early diagnosis at these unusual sites is crucial in patients with ET as thrombosis significantly affects the disease outcome and is associated with severe organ damage and high mortality [3].

## Case presentation

A 54-year-old female, known case of diabetes mellitus, hypertension, coronary artery disease, and seizure disorder presented with a complaint of pain in the left upper abdomen for the preceding five days. The pain was intermittent, moderate in intensity along with radiation to the back. There was no association of abdominal pain with eating. It was not associated with nausea, vomiting, or diarrhea. On examination, the patient was conscious, oriented, afebrile and her vitals were in the normal range. On abdominal examination, there was tenderness in the left hypochondrium, and the spleen was palpable four centimeters (cm) below the costal margin. Bowel sounds were normal and there were no signs of peritoneal inflammation. Cardiovascular, respiratory, and neurological examinations were unremarkable.

**Figure 1:**
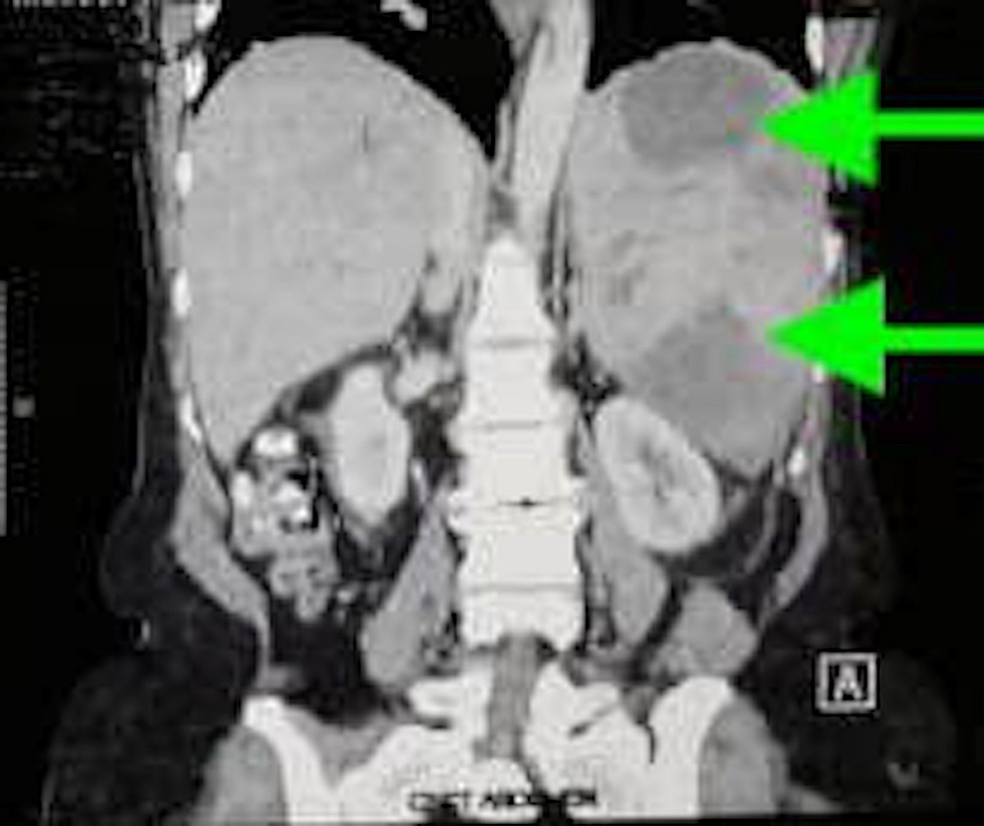
CECT of the abdomen showing hepatosplenomegaly with few irregular hypodense areas in the spleen suggestive of infarction (green arrows). CECT: Contrast-enhanced CT.

Investigations revealed thrombocytosis (1101 x 10^9^/L), leukocytosis (33.7 x 10^9^/L), hemoconcentration with hemoglobin of 12.5 g/dL, and a hematocrit of 48%. Peripheral blood film examination revealed anisopoikilocytosis, moderate hypochromia, leukocytosis, and thrombocytosis. There was no peripheral evidence of myelofibrosis. Renal and liver function tests were normal. Ultrasound abdomen was suggestive of splenomegaly (17.7 x 7 cm) along with splenic abscesses and portal vein thrombosis. The findings were confirmed on a contrast-enhanced CT (CECT) scan of the abdomen which demonstrated hepatomegaly with acute splenic and portal venous thrombosis with massive splenomegaly with areas indicating infarcts (Figure 1).

**Figure 2:**
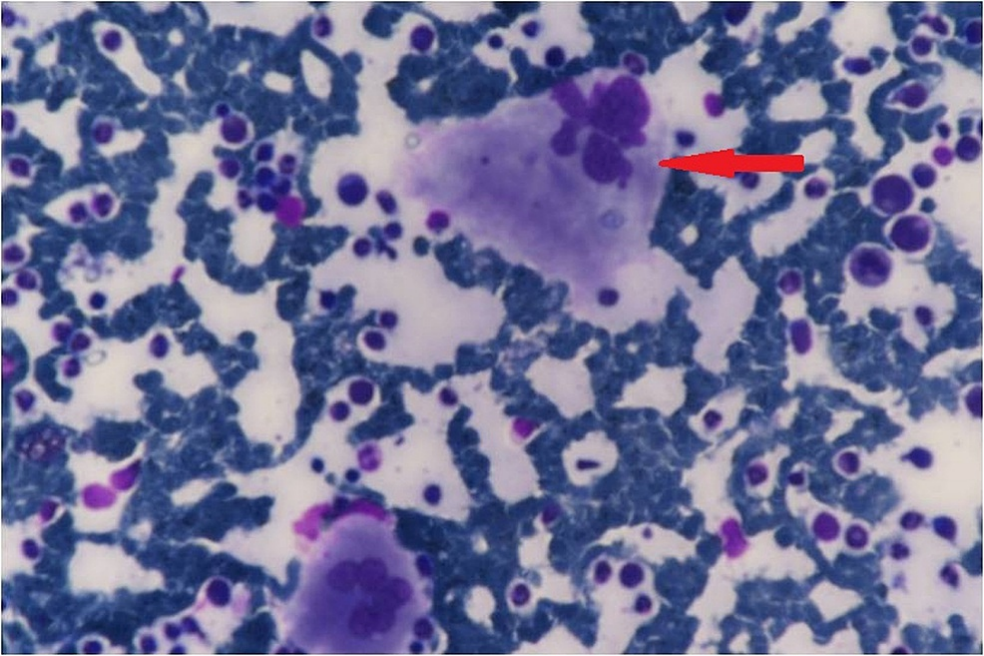
Bone marrow examination revealing a hypercellular marrow and megakaryocytic hyperplasia with staghorn nuclei (red arrow).

Upper GI endoscopy revealed grade one esophageal varices. The stomach and duodenum appeared normal. Viral markers, antinuclear antibody panel, and tumor markers including cancer antigen 19-9 (CA 19-9), carcinoembryonic antigen (CEA), and alpha-fetoprotein (AFP) were negative. Transient elastography of the liver (FibroScan) was not suggestive of cirrhosis. Two-dimensional (2D) echocardiography revealed a normal left ventricular systolic function and no regional wall motion abnormalities or evidence of any clot or vegetation. Bone marrow examination revealed hypercellular marrow (80-85% cellularity), megakaryocytic hyperplasia with focal clustering, and the presence of staghorn nuclei. These changes were suggestive of a myeloproliferative neoplasm, possibly ET (Figure 2).

A molecular study revealed JAK2V617F mutation positivity and was negative for BCR-ABL, thrombopoietin receptor gene (myeloproliferative leukemia, MPL), and calreticulin (CALR gene) mutations.

A diagnosis of ET (high-risk disease) was made and the patient was started on hydroxyurea. Portal vein thrombosis was managed with heparin followed by warfarin with international normalized ratio (INR) monitoring daily while the patient was admitted and every three to five days after discharge. INR was maintained between two and three. On follow-up evaluation six weeks later, she was asymptomatic with a platelet count of 95 x 10^9^/L and a total leucocyte count of 4.5 x 10^9^/L.

## Discussion

Mesenteric venous thrombosis (MVT) involving mesenteric, portal, and splenic veins is a common pathology with an array of clinical manifestations. Risk factors include inflammatory diseases such as pancreatitis and inflammatory bowel disease, postoperative states, cirrhosis, antiphospholipid syndrome, paroxysmal nocturnal hemoglobinuria, malignancy, oral contraceptives, pregnancy, and the postpartum period [4,5]. Hematological conditions involving thrombophilia should also be kept under consideration in the evaluation of thrombotic episodes, such as prothrombin gene mutation and factor V Leiden gain-of-function mutations or hematopoietic system-related clonal disorders like MPD which include chronic myeloid leukemia (CML) and BCR-ABL negative disorders (polycythemia vera [PV], ET, and primary myelofibrosis [PMF]). BCR-ABL negative MPD is associated with a somatic mutation in JAK2 which has been identified in almost all cases of PV (>95% positive), in 50-70% of ET and 50-60% of PMF patients [6]. Therefore, JAK2 being a pathognomonic feature of chronic MPD cannot be used for identifying the subtype, consequently, bone marrow examination remains mandatory in the diagnostic evaluation. In ET, bone marrow is normocellular or minimally hypercellular with megakaryocytosis which appears mature, multilobulated, either dispersed throughout the section or in loose clusters. This is in contrast to tightly packed, bizarre megakaryocytes seen in PMF with reticulin and collagen fibrosis [7,8]. Elevated inflammatory markers, such as ferritin, C-reactive protein (CRP), and erythrocyte sedimentation rate (ESR) with normal lactate dehydrogenase levels are seen in case of secondary thrombocytosis; however, these markers cannot be considered discriminatory at all as they can also be seen in ET.

ET clinically presents with vascular occlusive events and hemorrhages with transformation to leukemia or other myeloid diseases as a late complication. ET presenting with abdominal vein thrombosis is rare. In a study involving 460 patients with ET, Gangat et al. reported a prevalence rate of 4% for abdominal vein thrombosis [9]. In an acute variant, thrombocytosis and hyperviscosity lead to an increased thrombotic tendency. However, at later stages, functional alteration in platelet activity usually dominates.

ET usually has a good prognosis with regards to median survival. MVT is a poor prognostic marker due to leukemic or fibrotic transformation and hepatic failure [9]. Since there is no curative or disease-modifying therapy available currently, treatment strategies for ET include antiplatelet agents and cytoreductive therapy such as hydroxyurea, busulfan, anagrelide, and pegylated interferon-alfa. MPD-related variceal bleed is managed in the same way as due to cirrhosis or cancer, but the prognosis is comparatively better in the former case [10].

## Conclusions

Our case highlights the fact that patients with a history of thrombosis should be carefully evaluated for underlying MPD particularly if they show even slightly raised platelet or leukocyte counts. Early diagnosis is crucial in patients with ET as the development of thrombosis significantly affects morbidity and mortality. If diagnosed early, before the development of complications, patients can expect a good life expectancy with currently available therapy and have a low risk of future leukemic transformation or bone marrow fibrosis.
